# Associations Between Neighborhood Racialized Economic Segregation with Cardiometabolic Health and Cortisol in a Racially/Ethnically Diverse Sample of Children from Minneapolis—St. Paul

**DOI:** 10.1089/heq.2023.0246

**Published:** 2024-06-13

**Authors:** Christopher P. Carr, Allan D. Tate, Amanda Trofholz, Junia N. de Brito, Andrea N. Trejo, Michael F. Troy, Jerica M. Berge, Alicia Kunin-Batson

**Affiliations:** ^1^ Epidemiology and Biostatistics, College of Public Health, University of Georgia, Athens, Georgia, USA.; ^2^ Department of Family Medicine and Community Health, University of Minnesota, Minneapolis, Minnesota, USA.; ^3^ Department of Human Development and Family Science, University of Georgia, Athens, Georgia, USA.; ^4^ Children’s Minnesota, Minneapolis, Minnesota, USA.; ^5^ Department of Pediatrics and Center for Pediatric Obesity Medicine, University of Minnesota Medical School, Minneapolis, Minnesota, USA.; ^6^ Department of Family Medicine and Adult and Child Center for Outcomes Research and Delivery Science, University of Colorado, Aurora, Colorado, USA.

**Keywords:** health disparities, structural racism, childhood, cardiometabolic health

## Abstract

**Introduction:**

Past research shows that structural racism contributes to disparities in cardiometabolic health among racially/ethnically minoritized populations.

**Methods:**

This cross-sectional study examined the correlation between census tract-level racialized economic segregation and child health metrics among a racially and ethnically diverse cohort of 350 children (ages 6.5–13.8) from Minneapolis—St. Paul, MN.

**Results:**

A consistent cardiometabolic and cortisol outcome gradient was observed across the index of concentration at the extremes tertiles, such that health risk factors increased as tract privilege decreased.

**Conclusion:**

Racialized economic segregation was associated with less favorable child health outcomes, underscoring the potential importance of place-based interventions for promoting children’s health.

## Introduction

A robust literature shows longstanding disparities in cardiometabolic health (CMH) among racial/ethnic minoritized populations in the United States.^1,2^ A fundamental cause of health disparities is structural racism; defined as the systemic manifestation of racism via laws, institutional practices, and public policy.^3–5^ At the neighborhood level, structural racism may manifest as inequities resulting from policies and practices of disinvestment and exclusion of Black, Indigenous, and people of color (BIPOC). Past studies have shown that neighborhood factors are related to child health and health disparities.^6,7^ Chronic physiological stress, often measured through cortisol, has been identified as a mechanism through which structural racism may result in racial/ethnic CMH disparities.^8,9^ That is, discrimination and structural oppression may contribute to allostatic overload resulting in cardiovascular damage.^10^

Recent structural racism literature has increasingly utilized a measure known as the index of concentration at the extremes (ICE).^11,12^ ICE is a tool for measuring neighborhood-level racialized economic segregation through the quantification of neighborhood resource allocation and consequent privilege and oppression and has been shown to be effective for quantifying homogeneity at small geographic levels relative to other measures.^12^ In addition, the American Heart Association recently recommended further study into how structural factors affect CMH, specifically recognizing ICE as a valid classification of residential segregation and place-based inequity.^13^

The current study contributes to the literature by identifying structural racism as an important intervention target to improve population health and reduce health inequities. This study is shaped by ecosocial theory which postulates that racism contributes to racial/ethnic social inequities which are expressed as racial/ethnic health disparities.^14^ Recent calls to the field have recommended increased use of descriptive epidemiology to highlight persistent disparities related to structural racism, and this brief answers how place-based inequality is descriptively related to CMH in a racially and ethnically diverse sample from Minneapolis—St. Paul.^15^ We hypothesized that greater neighborhood inequities would be associated with poorer CMH and higher hair cortisol levels among a racially and ethnically diverse cohort of children in the Minneapolis—St. Paul, MN area.

## Materials and Methods

This is a descriptive secondary analysis of cross-sectional data from The *Family Matters* Study, a longitudinal observational cohort study examining risk and protective factors for cardiovascular health in children.^16^ The *Family Matters* Study recruited families with children between the ages of 5–9 years old (*n* = 1307) from primary care clinics in the Minneapolis—St. Paul area. Children with dietary restrictions, body mass index (BMI) < 5th percentile, and diagnosis of serious and persistent mental illness were excluded from enrollment. Researchers intentionally recruited families from six racial and ethnic groups: White, Black, Native American, Hmong, Somali/Ethiopian, and Latinx. Parents self-identified their own race and ethnicity, as well as that of their child on a questionnaire. About half of the families participated in an ecological momentary assessment (EMA) sub-study (*n* = 631) as detailed elsewhere.^17^ Analyses for the current investigation involved 350 children (55% of those eligible from the EMA sub-study) who participated in an in-person clinic visit (August 12th, 2019, through January 1st, 2022). During the clinic visit, the children provided a hair sample for cortisol assay and had trained study staff measure their height, weight, pulse rate, and blood pressure according to the standardized measurement protocol. All study protocols were approved by the Institutional Review Board at the University of Minnesota (1107S02666).

Staff obtained child height and weight via direct anthropometric measurement. STATA’s Zanthro procedure calculated the BMI percentile using the 2000 CDC Growth Reference. Study staff measured participant blood pressure in millimeters of mercury (mmHg) using an automated blood pressure device. Participants sat quietly without their legs crossed for 4–5 min before the first measure. Participants had three consecutive blood pressure measurements and the final two of three blood pressure measurements were averaged. Hair samples (3 cm in length) were taken from the posterior vertex of the child’s head for cortisol assay. A commercially available immunoassay with chemiluminescence detection (CLIA, IBL-Hamburg, Germany) determined cortisol concentrations following extraction. Hair cortisol values were natural log-transformed to address positive skewness and minimize outlier influence. We did not obtain hair samples from approximately 26% (*n* = 90) of children due to participant refusal or lack of availability (e.g., shaved head).

Three ICE measures at the census tract level were operationalized: (1) non-Hispanic White versus non-Hispanic Black homogeneity (WH/BL), (2) high (>$100,000) versus low-income homogeneity (<$25,000) (HI/LI), and (3) non-Hispanic White, high income versus non-Hispanic Black, low-income homogeneity (WH-HI/BL-LI). These three ICE variables are measures of racial and economic segregation and a combination of the two. We selected White vs. Black measures because it is the most prevalent form of racial segregation in the United States.^12^ We matched participant data to their respective census tract via their household address. We constructed these ICE measures using American Community Survey (ACS) data from 2014–2018, which preceded data collection.^18^ A total of 191 census tracts were included in the computation of ICE measures. For this analysis, we categorized the ICE measures into tertiles.

## Results

The analytic sample included 350 parent–child dyads (Table 1). Most caregivers (92%) and approximately half the children (53%) were female. Children were 9.8 years old on average (SD = 1.4), ranging from 6.5 to 13.8 years old. Forty-three percent of parents had a high school degree or some high school education, and 59% of households had an annual income of $49,999 or less. Nineteen percent of the children were from immigrant families. The EMA sample (*n* = 631) and the clinic sample (*n* = 350) had similar sociodemographic characteristics, with the exception of higher levels of household education and lower representation of Somali/Ethiopian participants among those who participated in the clinic visit.

**Table 1.** Parent and Child Participant Characteristics (*n* = 350)

Child female	185 (53%)
Child age in years (SD)	9.8 (1.4)
Child race/ethnicity	
White	106 (30%)
Black or African American	71 (20%)
Hispanic or Latin	36 (10%)
Asian American	37 (11%)
Native Hawaiian or other Pacific Islander	1 (0.3%)
American Indian or Native American	36 (10%)
Other	0 (0%)
Multiracial	63 (18%)
Parent born in the United States	282 (81%)
Parent race/ethnicity	
White	120 (34%)
Black or African American	77 (22%)
Hispanic or Latinx	37 (11%)
Asian American	38 (11%)
Native Hawaiian or other Pacific Islander	2 (1%)
American Indian or Native American	48 (14%)
Other	1 (0.3%)
Multiracial	27 (8%)
Parent educational attainment	
Some high school	21 (6%)
High school or associates	128 (37%)
Some college or bachelors	122 (35%)
Graduate degree	79 (23%)
Household income	
Less than $20,000	78 (22%)
$20,000–$34,999	74 (21%)
$35,000–$49,999	55 (16%)
$50,000–$74,999	43 (12%)
$75,000–$99,999	28 (8%)
$100,000 or more	68 (19%)
Not reported	4 (1%)

The analytic sample represented 156 census tracts in the Minneapolis—St. Paul area. Median household income and median property values within the predominantly Black census tracts (least privileged) were $44,649 and $114,357 less than the predominantly White (privileged) census tracts, respectively. Owner-occupied housing was 34% lower, and the poverty rate was 27% higher in predominantly Black census tracts compared with predominantly White census tracts. Measurement completeness was 100% for body mass index percentile (BMI%ile), 98% for pulse and blood pressure, and 74% for hair cortisol.

Primary outcomes included children’s CMH measures and hair cortisol across three operationalizations of ICE (Table 2). A consistent cardiometabolic and cortisol outcome gradient was observed across the ICE tertiles. That is, BMI%ile increased as tract privilege decreased. Among the WH/BL ICE variable, the average BMI%ile among the least privileged was 14.7 percentage points (pp) higher than the most privileged (75.2 ± 26 vs. 60.5 ± 29.1). Additional measures of CMH showed that the least privileged had, on average, higher pulse rates, systolic blood pressure (SBP), and diastolic blood pressure (DBP) across all ICE operationalizations. Hair cortisol results were also higher among least privileged tracts compared with tracts with concentrated privilege, especially among the ICE representation of economic inequity. Log-transformed hair cortisol levels among the HI/LI ICE variable showed that the least privileged had higher hair cortisol levels than the most privileged (log-transformed 2.6 ± 1.9 vs. 1.2 ± 1.4). Hair cortisol levels among WH-HI/BL-LI ICE variables showed that the least privileged had higher hair cortisol levels than the most privileged group (log-transformed 2.5 ± 1.8 vs. 1.2 ± 1.4).

**Table 2.** Racialized Economic Segregation Correlates of Cardiometabolic Outcomes in Children-8/12/19 to 1/31/22 Minneapolis, MN

	Cardiometabolic and Cortisol Outcomes				
	Pulse average (bpm) (*n* = 344)	SBP (mmHg) (*n* = 344)	DBP (mmHg) (*n* = 344)	BMI%ile (*n* = 350)	Hair cortisol (log-transformed) (*n* = 260)
White extreme/ black extreme (census tracts = 191)	Mean ± SD				
(1) Concentrated deprivation	89.0 ± 12.1	106.8 ± 12.3	63.2 ± 8.1	75.2 ± 26.0	2.4 ± 1.8
(2) Middle	85.0 ± 11.5	104.0 ± 10.9	60.8 ± 6.3	70.2 ± 28.4	2.0 ± 1.5
(3) Concentrated privileged	85.2 ± 10.0	100.9 ± 8.7	59.3 ± 6.5	60.5 ± 29.1	1.1 ± 1.3
High-income extreme/ Low-income extreme					
(1) Concentrated deprivation	89.3 ± 12.5	106.9 ± 11.8	63.3 ± 7.8	76.2 ± 25.8	2.6 ± 1.9
(2) Middle	84.4 ± 10.8	103.2 ± 11.4	60.6 ± 6.8	66.5 ± 29.6	1.7 ± 1.5
(3) Concentrated privileged	85.6 ± 10.1	101.6 ± 8.9	59.5 ± 6.4	63.3 ± 28.5	1.2 ± 1.4
White, high-income extreme/Black, low-income extreme					
(1) Concentrated deprivation	88.9 ± 12.8	107.0 ± 12.3	63.6 ± 7.9	75.2 ± 26.7	2.5 ± 1.8
(2) Middle	84.9 ± 10.4	103.3 ± 10.5	60.5 ± 6.6	68.7 ± 28.2	1.7 ± 1.5
(3) Concentrated privileged	85.3 ± 10.3	101.4 ± 9.1	59.3 ± 6.2	61.9 ± 29.1	1.2 ± 1.4

Sample Interpretation: In the column labeled BMI%ile and the White Extreme Black Extreme row, those at concentrated deprivation (1) had a mean BMI%ile of 75.2 which is 14.7 percentage points higher than the score at the concentrated privileged (3) which is 60.5. bpm, beats per minute; SBP, systolic blood pressure; DBP, diastolic blood pressure; BMI%ile, BMI percentile.

## Discussion

The current study illustrates how racialized segregation and economic opportunities at the census tract-level are associated with children’s CMH and cortisol levels. Specifically, the use of multiple operationalizations of ICE demonstrated that tract racial segregation, economic inequity, and the intersection of the two are associated with less favorable child health metrics among children ages 6.5 to 13.8, a developmental stage at which cardiometabolic risk factors can be detected but before disease manifests. Given the cross-sectional nature of this analysis, it cannot be determined whether the associations between racialized economic segregation and health outcomes differ across life course or vary by developmental stage. Systemic strategies for promoting population-level CMH are needed to promote equitable health outcomes throughout the life course.^19,20^ The economic differences presented between the sample census tracts highlight specific geographic areas for community reinvestment, such as housing quality or programs that convert renters into homeowners.

The strengths of this study include using ICE as a measure of racialized economic segregation alongside in-clinic measures of children’s CMH and hair cortisol, which is not subject to situational variability and allows for an estimate of HPA-axis activation over an extended period of time. Limitations include the cross-sectional nature of the data, the lack of information available about potential confounders of cortisol levels (e.g., child corticosteroid use), and the descriptive nature of this analysis, which does not consider the role of other potential contributors to children’s CMH. Future studies should examine the contribution of ICE with other important contributors to children’s CMH, including individual and family level variables (e.g., household income, parent education), and other systems that reinforce structural racism (e.g., access to quality health care, affordable housing).

## Conclusion

Child CMH and hair cortisol levels showed an association with census tract racial and economic disadvantages, underscoring the potential importance of place-based interventions for promoting children’s health. Longitudinal, prospective studies are needed to better understand the role of multilevel structural determinants in children’s health outcomes.

## Abbreviation Used

ACS: American Community Survey
BIPOC: Black, Indigenous, and people of color
BMI: Body mass index
BMI%ile: Body mass index percentile
CMH: Cardiometabolic health
DBP: Diastolic blood pressure
EMA: Ecological momentary assessment
HI/LI: High (>$100,000) versus low-income homogeneity (<$25,000)
ICE: Index of concentration at the extremes
mmHg: Millimeters of mercury
PP: Percentage points
SBP: Systolic blood pressure
WH/BL: non-Hispanic White versus non-Hispanic Black homogeneity
WH-HI/BL-LI: non-Hispanic White, high income versus non-Hispanic Black, low-income homogeneity

## References

[1] National Center for Health Statistics (US). Health, United States, 2015: With Special Feature on Racial and Ethnic Health Disparities. Health, United States. National Center for Health Statistics (US): Hyattsville (MD); 2016. 27308685

[2] Puckrein GA, Egan BM, Howard G. Social and medical determinants of cardiometabolic health: The big picture. Ethn Dis 2015;25(4):521–524; doi: 10.18865/ed.25.4.521 n.d 26673674 PMC4671441

[3] Phelan JC, Link BG. Is racism a fundamental cause of inequalities in health? Annu Rev Sociol 2015;41(1):311–330; doi: 10.1146/annurev-soc-073014-112305

[4] Riley AR. Neighborhood disadvantage, residential segregation, and beyond-lessons for studying structural racism and health. J Racial Ethn Health Disparities 2018;5(2):357–365; doi: 10.1007/s40615-017-0378-5 28573643

[5] Lynch EE, Malcoe LH, Laurent SE, et al. The legacy of structural racism: Associations between historic redlining, current mortgage lending, and health. SSM Popul Health 2021;14:100793; doi: 10.1016/j.ssmph.2021.100793 33997243 PMC8099638

[6] Choi J-K, Kelley M, Wang D, et al. Anonymous. Neighborhood environment and child health in immigrant families: Using nationally representative individual, family, and community datasets. Am J Health Promot 2021;35(7):948–956; Available from: https://journals.sagepub.com/doi/abs/10.1177/08901171211012522 [Last accessed: March 16, 2024] 33906427 10.1177/08901171211012522

[7] Wang G, Schwartz GL, Kershaw KN, et al. The association of residential racial segregation with health among U.S. children: A nationwide longitudinal Study. SSM Popul Health 2022;19:101250; doi: 10.1016/j.ssmph.2022.101250 36238814 PMC9550534

[8] DeSantis AS, Adam EK, Doane LD, et al. Racial/ethnic differences in cortisol diurnal rhythms in a community sample of adolescents. J Adolesc Health 2007;41(1):3–13; doi: 10.1016/j.jadohealth.2007.03.006 17577528

[9] Saban KL, Mathews HL, DeVon HA, et al. Epigenetics and social context: Implications for disparity in cardiovascular disease. Aging Dis 2014;5(5):346–355; doi: 10.14336/AD.2014.0500346 25276493 PMC4173800

[10] Panza GA, Puhl RM, Taylor BA, et al. Links between discrimination and cardiovascular health among socially stigmatized groups: A systematic Review. PLoS ONE 2019;14(6):e0217623; doi: 10.1371/journal.pone.0217623 31181102 PMC6557496

[11] Krieger N, Waterman PD, Spasojevic J, et al. Public health monitoring of privilege and deprivation with the index of concentration at the extremes. Am J Public Health 2016;106(2):256–263; doi: 10.2105/AJPH.2015.302955 26691119 PMC4815605

[12] Krieger N, Kim R, Feldman J, et al. Using the Index of concentration at the extremes at multiple geographical levels to monitor health inequities in an era of growing spatial social polarization: Massachusetts, USA (2010-14). Int J Epidemiol 2018;47(3):788–819; doi: 10.1093/ije/dyy004 29522187

[13] Kershaw KN, Magnani JW, Diez Roux AV, et al. Neighborhoods and cardiovascular health: A scientific statement from the American Heart Association. Circ Cardiovasc Qual Outcomes 2024;17(1):e000124; doi: 10.1161/HCQ.0000000000000124 38073532

[14] Krieger N. Methods for the scientific Study of discrimination and health: An ecosocial approach. Am J Public Health 2012;102(5):936–944; doi: 10.2105/AJPH.2011.300544 22420803 PMC3484783

[15] Fox MP, Murray EJ, Lesko CR, et al. On the need to revitalize descriptive epidemiology. American Journal of Epidemiology 2022;191(7):1174–1179; doi: 10.1093/aje/kwac056 35325036 PMC9383568

[16] Berge JM, Trofholz A, Tate AD, et al. Examining unanswered questions about the home environment and childhood obesity disparities using an incremental, mixed-methods, longitudinal study design: The family matters Study. Contemp Clin Trials 2017;62:61–76; doi: 10.1016/j.cct.2017.08.002 28800894 PMC5641262

[17] Berge JM, Tate A, Trofholz A, et al. Momentary parental stress and food-related parenting practices. Pediatrics 2017;140(6):e20172295; doi: 10.1542/peds.2017-2295 29167378 PMC5703772

[18] U.S. Census Bureau. American Community Survey 2014-2018 5-Year Estimates. 2018.

[19] Brown AF, Ma GX, Miranda J, et al. Structural interventions to reduce and eliminate health disparities. Am J Public Health 2019;109(S1):S72–S78; doi: 10.2105/AJPH.2018.304844 30699019 PMC6356131

[20] Bailey ZD, Krieger N, Agénor M, et al. Structural racism and health inequities in the USA: Evidence and interventions. Lancet 2017;389(10077):1453–1463; doi: 10.1016/S0140-6736(17)30569-X 28402827

